# Hemoadsorption in critically ill neonatal and pediatric patients: a retrospective cohort study from a Latin American tertiary center

**DOI:** 10.3389/fmed.2025.1650118

**Published:** 2025-09-25

**Authors:** Byron Enrique Piñeres-Olave, Jhojan Sebastian Herrera-Vargas, Daniel Tibaduiza, Alejandro Marin-Agudelo, María Piedad Sarmiento, Sandra Salazar, Laura Niño-Jaimes, Natalia Arango-Mesa

**Affiliations:** ^1^Pediatric Critical Care Unit, Somer Clinic, Rionegro, Colombia; ^2^Research Department, Somer Clinic, Rionegro, Colombia; ^3^Research Department, Somer Incare Cardiovascular Center, Rionegro, Colombia

**Keywords:** neonatal and pediatric patients, septic shock, ARDS, CRRT, ECMO, hemoadsorption, CytoSorb

## Abstract

**Introduction:**

Extracorporeal blood purification therapies, such as hemoadsorption, are increasingly utilized in intensive care to modulate inflammation, improve organ function, and reduce vasoactive support. However, data on their use in neonatal and pediatric populations remain limited, particularly in low-resource settings. This study aimed to evaluate clinical and laboratory outcomes in critically ill pediatric patients receiving hemoadsorption therapy alongside extracorporeal organ support.

**Methods:**

We conducted a single-center retrospective cohort study in a tertiary neonatal and pediatric intensive care unit in Latin America. Eleven critically ill patients received hemoadsorption using CytoSorb^®^ or Oxiris^®^ in combination with continuous renal replacement therapy (CRRT) and/or extracorporeal membrane oxygenation (ECMO). We assessed organ dysfunction scores (PELOD-2), vasoactive-inotropic score (VIS), inflammatory markers (CRP, lactate), oxygenation parameters, and liver and hematologic profiles before and after therapy.

**Results:**

Hemoadsorption was associated with reductions in PELOD-2 (median 11–7; *p* = 0.036) and VIS (median 75–6; *p* = 0.014). Lactate levels decreased significantly (*p* < 0.001), and oxygenation improved (PaO₂/FiO₂, median 69–104; *p* = 0.042). CRP levels declined without reaching statistical significance. Liver and hematologic markers remained largely unchanged. 28-day-mortality was 54.5%.

**Discussion:**

Hemoadsorption in conjunction with CRRT and/or ECMO showed potential to improve hemodynamic stability, oxygenation, and inflammation in critically ill pediatric patients. These findings support further investigation of hemoadsorption as an adjunctive therapy in pediatric critical care, especially in resource-limited environments.

## Graphical abstract

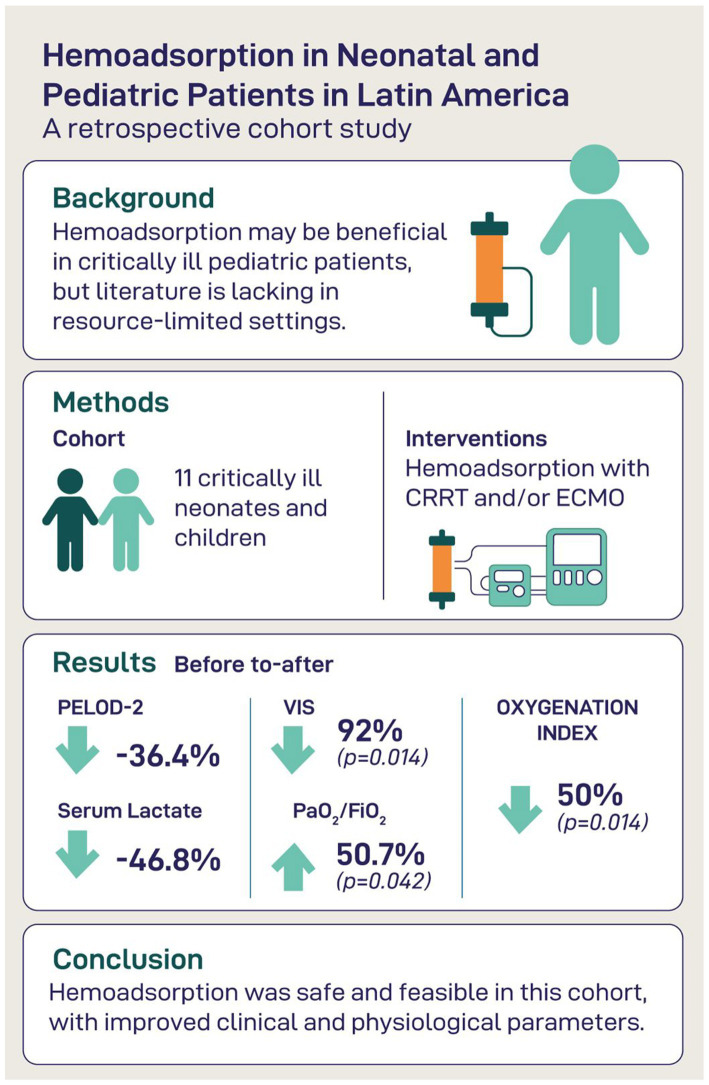

## Introduction

Neonatal and pediatric patients admitted to intensive care units often face life-threatening conditions that require highly specialized care (1), which is particularly challenging in settings with inadequate resources (2, 3). Among the most challenging scenarios is critical illness associated with severe infection, systemic inflammation, and multiple organ dysfunction syndrome (MODS). In this vulnerable population, sepsis remains a leading cause of morbidity and mortality, triggering a cascade of inflammatory and immunologic responses that can rapidly overwhelm the child’s physiological reserves (4).

To support failing organs and maintain homeostasis in such complex clinical situations, extracorporeal life support (ECLS) techniques such as continuous renal replacement therapy (CRRT) and extracorporeal membrane oxygenation (ECMO) are increasingly employed in pediatric intensive care settings (5, 6). These modalities not only provide vital organ support but also serve as platforms for adjunctive therapies aimed at modulating the host’s pathophysiological response to critical illness (7).

Among these adjunctive strategies, hemoadsorption therapy has emerged as a promising tool. Hemoadsorption involves the extracorporeal removal of circulating inflammatory mediators, bacterial toxins, and other pathogenic substances that are often not adequately cleared by conventional dialysis or filtration techniques due to their molecular size, lipophilicity, or protein-binding characteristics (8). By incorporating sorbent cartridges into the extracorporeal circuit, hemoadsorption may help to mitigate the excessive inflammatory response and contribute to hemodynamic stabilization in severely ill patients (9).

Among the available hemoadsorption technologies, CytoSorb^®^ and Oxiris^®^ are two devices commonly used in clinical practice for the extracorporeal removal of inflammatory mediators and endotoxins. CytoSorb^®^ employs biocompatible polystyrene divinylbenzene polymer beads designed to adsorb a wide spectrum of middle molecular weight substances, including cytokines and other pro-inflammatory mediators (10). Oxiris^®^, on the other hand, is a hemodialysis filter with adsorptive properties capable of endotoxin and cytokine removal, making it suitable for patients with both renal failure and systemic inflammation (11).

Despite growing interest and adoption of these technologies in adult critical care, data on their use in neonates and children remain sparse. While most available data remain limited to small case series and observational studies, a recently published single-arm interventional study by Bottari et al. has contributed initial evidence toward addressing these gaps, although key questions around optimal timing, dosing, and long-term outcomes require further clarification (12). Moreover, larger pediatric studies are needed to confirm these preliminary findings. There is a particular need to characterize the safety, feasibility, and potential impact of hemoadsorption in resource-limited settings, where advanced supportive therapies are less accessible.

This study aims to address this gap by describing clinical and laboratory trends in a cohort of critically ill neonatal and pediatric patients treated with hemoadsorption in a high-complexity intensive care unit in Latin America. By focusing on a real-world population already receiving ECMO and/or CRRT, we explore the adjunctive use of CytoSorb^®^ and Oxiris^®^ in the management of severe inflammatory states and multiple organ failure in children.

## Materials and methods

This retrospective, observational study was conducted in a high-complexity Pediatric Intensive Care Unit (PICU) between June and December 2024. This PICU is a medical-surgical unit specializing in cardiovascular surgery for congenital heart disease, ECMO support, and the care of critically ill patients from across the country. The study was approved by the Institutional Review Board (IRB) of Somer Clinic, Rionegro, Colombia. Following IRB/IEC approval, informed consent was obtained from the patients’ legal representatives before any data collection. The study was conducted in accordance with International Organization for Standardization (ISO) 14,155:2020, the Declaration of Helsinki and the standards of Good Clinical Practice.

Included were all eligible consecutive neonatal (0–28 days) and pediatric (1 month–18 years) patients who received hemoadsorption therapy during the study period, ensuring a cohort representative of real-world-experience. Enrolled patients were treated according to the departments’ routine standard of care.

**Figure 1:**
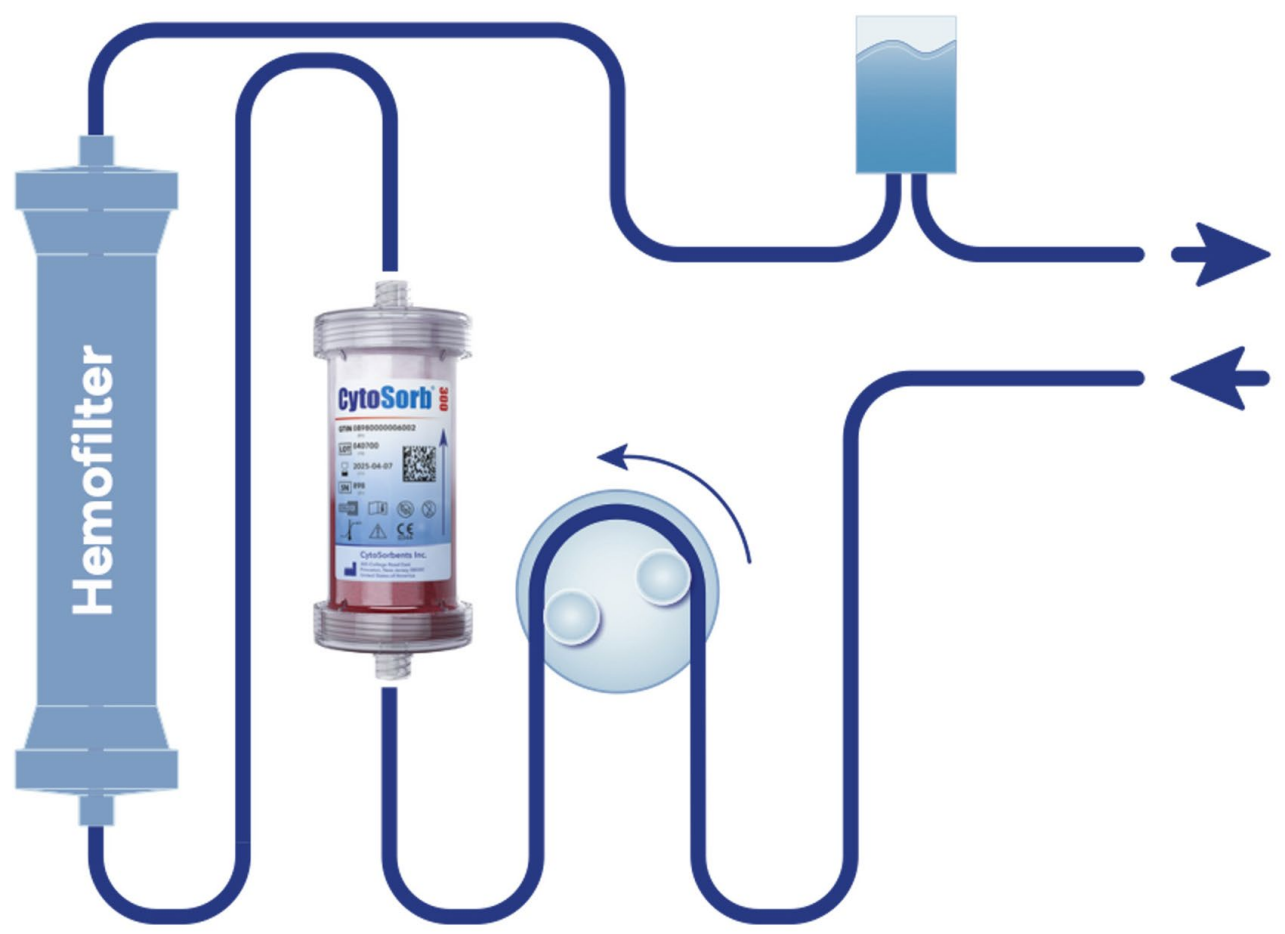
Integration of CytoSorb^®^ into continuous renal replacement therapy (CRRT) circuit with HF20 or ST60 hemofilter. Schematic representation of a pre-hemofilter setup in continuous veno-venous hemodiafiltration (CVVHDF) mode. The CytoSorb^®^ cartridge is positioned in the extracorporeal blood circuit upstream of the hemofilter (HF20 or ST60). This configuration was used in all patients in whom CytoSorb^®^ was applied in this study.

CRRT was performed using a Prismaflex device (Gambro, Germany) along with HF20 or ST60 filters run in continuous veno-venous hemofiltration (CVVHF) or continuous veno-venous hemodiafiltration (CVVHDF) mode and the hemoadsorber was placed pre-hemofilter (Figure 1). For ECMO-supported patients, two configurations were used: hemoadsorption with a CytoSorb column was integrated directly into the ECMO circuit, which was operated with a Trolley Machine (Sorin Medical^®^) at flows of 120–160 mL/kg/min, with the CytoSorb^®^ bridge running at 200 mL/min without additional anticoagulation. For patients treated with Oxiris, the filter was positioned in the CRRT circuit connected to the ECMO system (Cardiohelp^®^, Maquet Group), operated with ECMO blood flows of 100–120 mL/kg/min and CRRT blood flows (Qb) of 100 mL/min, with regional anticoagulation using citrate as per institutional protocol.

CytoSorb cartridges were maintained in the circuit until there was no further clinical improvement such as for example a continued decline in vasoactive support requirements (as represented by VIS) or when signs of circuit clotting necessitated replacement.

In the absence of standardized markers for cartridge saturation, replacement decisions were guided by clinical judgment, typically occurring within 24 h of initiation. Clinical and paraclinical data were obtained from electronic medical records and analyzed, considering variables including PELOD-2, Vasoactive-Inotropic Score (VIS), pH, PaO_2_/FiO_2_, oxygenation index (mean airway Pressure x PaO_2_/FiO_2_), C-reactive protein (CRP), lactate, bilirubin, leukocytes, as well as platelets. All these clinical and laboratory parameters were collected immediately before the initiation of hemoadsorption and at the time of its discontinuation.

Qualitative variables were described as absolute and relative frequencies, while quantitative variables were expressed as medians with interquartile ranges. The Wilcoxon test for paired samples was used for pre- and post-therapy comparisons, and the Mann–Whitney test for independent samples was used to assess the association with mortality, with a significance level of *p* < 0.05.

## Results

During the study period, 11 critically ill neonatal and pediatric patients received hemoadsorption therapy and were included in this analysis. The median age was 3.0 years (IQR: 0.63–10.00), and the median weight was 14.0 kg (IQR: 9.35–37.50), with 63.4% of the patients being male. All patients presented with multiple organ dysfunction of varying etiologies. Comorbidities were frequent and diverse, including acute kidney injury (AKI, KDIGO stage 2–3) in 10 patients (90.9%), acute respiratory distress syndrome (ARDS) in 6 (54.5%), oncologic disease in 3 (27.2%), and liver failure in one patient (9.1%), reflecting the complexity of the population managed in this high-acuity unit. The underlying diagnoses leading to hemoadsorption therapy were diverse and reflected a broad spectrum of critical illness in pediatric patients. The most common indication was septic shock, present in eight patients (72.7%). Among these, five patients (45.5%) fulfilled the definition of catecholamine-refractory septic shock, based on the European Society of Pediatric and Neonatal Intensive Care (ESPNIC) expert criteria (13). The underlying sources of infection among the eight patients with septic shock varied and included methicillin-sensitive *Staphylococcus aureus* (*n* = 3), viral and bacterial co-infection with COVID-19, *Streptococcus pneumoniae*, and *Haemophilus influenzae* (*n* = 1), *Klebsiella pneumoniae* (*n* = 1), extended-spectrum beta-lactamase-producing *Escherichia coli* (*n* = 1), *Metapneumovirus* (*n* = 1), and a case of bloodstream infection with *Pseudomonas aeruginosa* and septic arthritis due to methicillin-resistant *Staphylococcus aureus* (*n* = 1). Two patients (18.2%) required hemoadsorption following toxic exposure: one due to mercury poisoning associated with ARDS and acute kidney injury, and another due to paraquat ingestion. Finally, one patient (9.1%) received hemoadsorption for hyperbilirubinemia in the context of severe liver dysfunction.

In terms of extracorporeal support, all patients were already receiving either continuous renal replacement therapy (CRRT), extracorporeal membrane oxygenation (ECMO), or both at the time hemoadsorption was initiated. CytoSorb^®^ cartridges were used in 10 patients, while Oxiris^®^ was employed in one case. Hemoadsorption was performed directly via the ECMO circuit in one neonate; in all other patients, it was applied in conjunction with CRRT, or integrated into the ECMO-CRRT configuration (Table 1).

**Table 1:** Patient characteristics and treatment as well as outcome variables (*n* = 11).

Baseline	Median (IQR) or n (%)
Age (years)	3.00 (0.63–10.00)
Weight (kg)	14.00 (9.35–37.50)
Gender (male)	7 (63.64%)
Comorbidities	
AKI (KDIGO 2–3)	10 (90.9%)
Oncology	3 (27.2%)
ARDS	6 (54.5%)
Liver failure	1 (9.09%)
Diagnosis	
Septic shock	8 (72.72%)
- Methiciline sensitive S.aureus	3/8
- Covid19, *S. pneumoniae*, and *H. influenzae*	1/8
- *Klebsiella pneumoniae*	1/8
- *E. coli* BLEE+	1/8
- Metapneumovirus	1/8
- BSI P.aeruginosa and septic arthritis MRSA	1/8
Paraquat poisoning	1 (9.09%)
Mercury poisoning—ARDS	1 (9.09%)
Hyperbilirubinemia	1 (9.09%)
Indication for hemoadsorption therapy	
Refractory septic shock	5 (45.45%)
Toxic shock by MSSA	1 (9.09%)
Septic shock in ECMO	2 (18.18%)
Inhaled mercury poisoning	1 (9.09%)
Paraquat poisoning	1 (9.09%)
Hyperbilirubinemia	1 (9.09%)
Hemoadsorption therapy used	
Oxiris ^®^	1 (9.09%)
CytoSorb ^®^	10 (90.91%)
System for administration of hemoadsorption therapy	
Extracorporeal membrane oxygenation	1 (9.09%)
Continuous renal replacement therapy	10 (90.91%)
Modality of CRRT	
Continuous veno-venous hemodiafiltration	7 (70%)
Continuous veno-venous hemofiltration	3 (30%)
Additionally applied extracorporeal therapies	
ECMO alone	1 (9.09%)
CRRT alone	5 (45.45%)
ECMO + CRRT	5 (45.45%)
Supportive therapy durations	
Duration of CRRT (days)	6.5 (3.5–9.5)
Duration of hemoadsorption therapy (h)	30.00 (19.00–37.50)
Number of hemoadsorption columns used (n)	1.00 (1.00–1.50)
Duration of ECMO (days)	13.5 (9.75–18.75)
Duration of mechanical ventilation (days)	9.0 (8.0–12.0)
Duration of vasopressor support (days)	2.0 (1.0–3.0)
Outcome	
Length of stay in PICU (days)	14.00 (11.00–19.50)
Length of hospital stay (days)	23.00 (11.00–29.00)
PICU survival	45.45%
28-day survival	45.45%

Quantitative variables are presented as medians (interquartile range, IQR), while categorical variables are expressed as absolute frequency (percentage, %). ARDS, acute respiratory distress syndrome; AKI, acute kidney injury; BLEE+, extended-spectrum beta-lactamase; BSI, blood stream infection; CRRT, continuous renal replacement therapy; ECMO, extracorporeal membrane oxygenation; MRSA, methicillin-resistant *Staphylococcus aureus*; MSSA, methicillin-sensitive *Staphylococcus aureus*.

The median duration of hemoadsorption therapy was 30 h (IQR: 19.00–37.50). Mechanical ventilation was required in all patients, with a median duration of 9.0 days (IQR 8.0–12.0). Vasopressor support was required for a median of 2 days (IQR 1.0–3.0). The median PICU length of stay was 14.0 days (IQR: 11.0–19.5), and the total hospital stay was 23.0 days (IQR: 11.0–29.0). 28-day mortality was 54.5% which was equivalent to the PICU mortality, reflecting the critical nature of the underlying diseases in this cohort. Detailed patient characteristics and treatment variables are presented in Table 1. Moreover, given the small sample size and case-series design of this study, individual pre- and post-treatment data for each patient are presented in tabular form to allow for transparent evaluation of clinical and laboratory trends (Table 2).

**Table 2:** Individual clinical and laboratory parameters in pediatric patients undergoing hemoadsorption therapy.

Patient number	1	2	3	4	5	6	7	8	9	10	11
Weight (kg)	35	49	3.3	8.7	14	40	2.6	68	10	10	25
Indication for admission to PICU	Paraquat poisoning	Septic shock	Septic shock	Inhaled Mercury poisoning	Septic shock	Septic shock	Hyperbilirubinemia	Septic shock -	Septic shock	Septic shock	Septic shock
Type of infection	–	*MSSA*	*SARS CoV-2, S. pneumoniae, H. influenzae*	*-*	*MSSA*	*MSSA*	–	*K. pneumoniae*	*Metapneumovirus*	*E.coli BLEE+*	*P. aeruginosa and MRSA*
Indication for blood purification treatment	Paraquat poisoning	Toxic shock by MSSA	Refractory septic shock	Inhaled mercury poisoning	Septic shock in ECMO	Refractory septic shock	Hyperbilirubinemia	Refractory septic shock	Refractory septic shock	Refractory septic shock	Septic shock in ECMO
ARDS as comorbidity [Yes, No]	No	No	Yes	Yes	Yes	Yes	Yes	No	Yes	No	No
Hemoadsorption device used	Oxiris	Cytosorb	Cytosorb	Cytosorb	Cytosorb	Cytosorb	Cytosorb	Cytosorb	Cytosorb	Cytosorb	Cytosorb
Duration of blood purification											
- Hemoadsorption (hours)	87	36	8	45	39	21	29	7	17	30	33
- CRRT (days)	4	9	–	23	7	2	6	1	10	5	9
Mortality in PICU [yes, no]	No	Yes	Yes	Yes	No	No	Yes	Yes	No	No	Yes
Vasoactive index score											
- Pre	0	66	10	0	75	113	3	110	100	186	76
- Post	0	30	7	0	10	6	3	63	3	15	5
PELOD-2											
- Pre	9	14	10	12	4	8	8	17	14	11	13
- Post	6	7	10	12	4	2	8	17	4	7	9
Oxygenation index (mmHg)											
- pre	24	5	11	18	31	23	16	26	25	16	29
- post	7	4	9	17	23	6	20	24	22	6	12
PaO₂/FiO₂ (mmHg) [pre; post]											
- Pre	107	197	299	45	57	74	62	57	47	268	69
- Post	246	241	322	51	79	88	49	104	54	230	145
Lactate (mmol/l) [pre; post]											
- Pre	4.0	12.5	4.7	3.1	12.0	4.3	3.1	12.6	2.8	13.0	6.0
- Post	1.2	5.0	2.4	1.4	3.7	1.7	2.5	8.0	1.3	3.8	3.0
C-reactive protein (mg/L)											
- Pre	25	35	66	3	31	33	38	24	10	101	241
- Post	6	28	0.8	2	2	26	16	–	4	29	327
D-dimers [pre; post]											
- Pre	–	–	–	344	–	6,244	–	–	–	5,079	12,799
- Post	–	–	–	122	–	3,391	–	–	–	3,626	7,197
Leukocytes (cells/μL)											
- Pre	10,500	2,980	9,940	26,400	22,000	2,760	12,820	20	22,450	1850	3,190
- Post	8,840	2,310	7,450	11,510	10,800	11,810	14,500	–	15,300	310	9,790
Platelets (cells/μL)											
- Pre	213,000	10,000	132,000	109,000	170,000	92,000	312,000	3,000	12,000	37,000	76,000
- Post	111,000	45,000	122,000	232,000	96,000	67,000	21,000	–	148,000	133,000	89,000
Hemoglobin (g/dL)											
- Pre	10.9	11.6	12.4	10.4	10.6	9.7	12.9	8.0	13.3	8.4	9.1
- Post	9.8	11.9	11.8	11.1	12.5	10.2	10.2	–	13.0	9.1	9.9

ARDS, acute respiratory distress syndrome; BLEE+, extended-spectrum beta-lactamase, CRRT, continuous renal replacement therapy; ECMO, extracorporeal membrane oxygenation, MRSA, methicillin-resistant *Staphylococcus aureus*, MSSA, methicillin-sensitive Staphylococcus aureus; PaO₂/FiO₂, relationship between arterial oxygen pressure and fraction of inspired oxygen; PELOD 2, pediatric logistic organ dysfunction score 2.

### Clinical organ dysfunction scores

**Figure 2:**
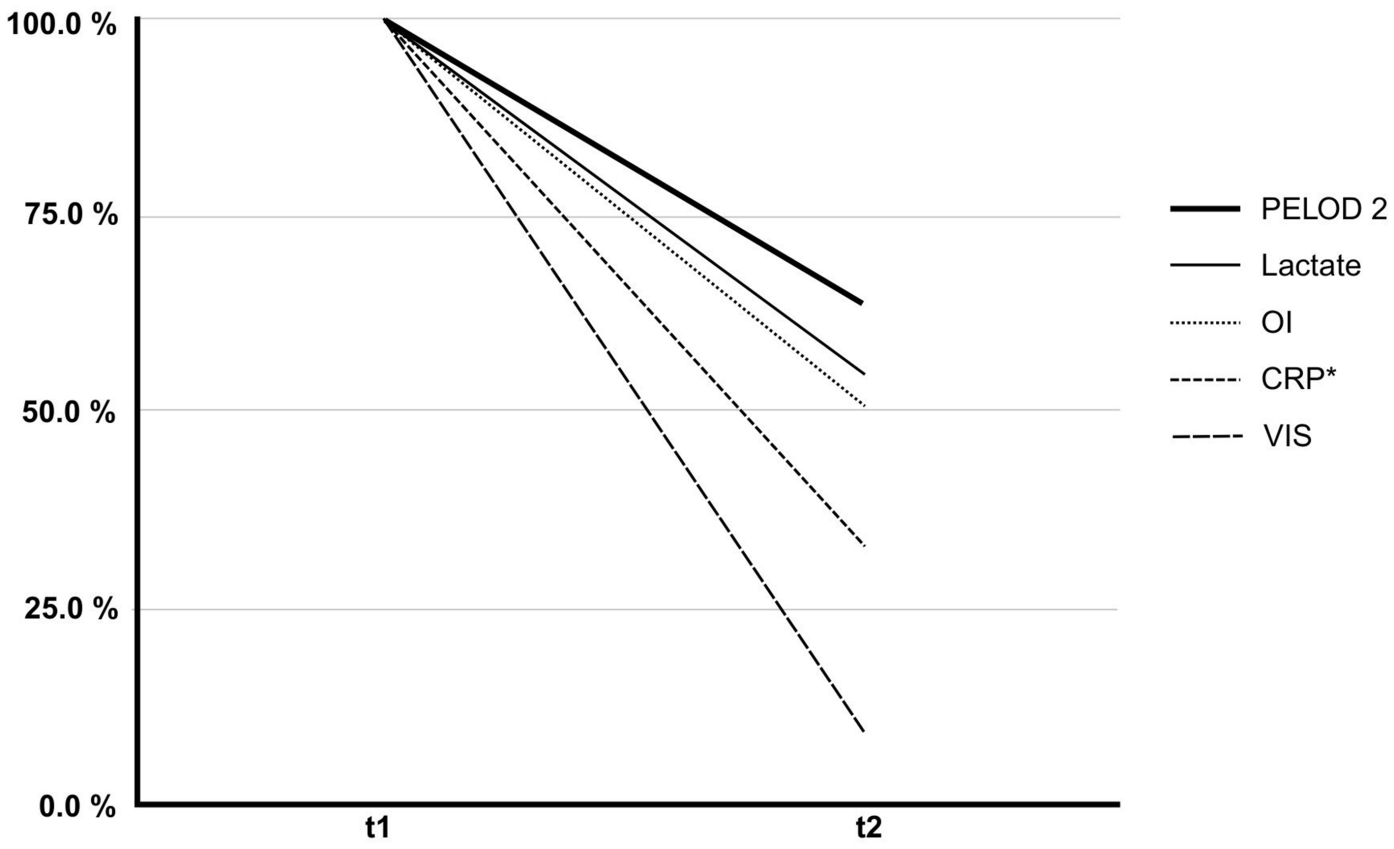
Percentage change in clinical and laboratory parameters before (t1) and after (t2) hemoadsorption therapy. The figure illustrates the relative reduction in PELOD-2 score, Vasoactive-Inotropic Score (VIS), C-reactive protein (CRP), serum lactate, and oxygenation index (OI) from initiation (t1) to discontinuation (t2) of hemoadsorption. Each parameter is presented as a percentage of its initial value at t1 (normalized to 100%). These changes reflect improvements in organ dysfunction (PELOD-2), hemodynamic support requirements (VIS), systemic inflammation (CRP), tissue perfusion (lactate), and pulmonary function (OI). * *n* = 10/11.

The initial median PELOD-2 score was 11 (IQR: 9–13), which significantly decreased to a median of 7 (IQR: 5–10) after discontinuation of treatment (*p* = 0.036) (Figure 2).

The VIS also significantly decreased from an initial median of 75–6 at the end of hemoadsorption therapy (*p* = 0.014), with a mean decrease of 80% (Figure 2). In the subgroup of patients with refractory septic shock (*n* = 5), vasoactive-inotropic scores (VIS) decreased following hemoadsorption therapy as follows: Patient 3: from 10 to 7; Patient 6: from 113 to 6, Patient 8: from 110 to 63, Patient 9: from 100 to 3, Patient 10: from 186 to 15 (Table 2).

### Inflammatory markers and perfusion

C-reactive protein (CRP) decreased from a median of 34–11 mg/dL at the end of therapy, representing a decrease of 67.6%, although not reaching statistical significance (*p* = 0.083) (Figure 2). Serum lactate, an indirect marker of tissue hypoperfusion, showed a significant decrease from 4.7 to 2.5 mmol/L (*p* < 0.001).

### Lung function and oxygenation

The PaO₂/FiO₂ ratio improved significantly after therapy, from a median of 69–104 mmHg (*p* = 0.042). In parallel, the oxygenation index (OI) decreased by 50% (*p* = 0.014) (Figure 2). Among the entire cohort, six patient fulfilled the PALICC-2 definition for pediatric acute respiratory distress syndrome (ARDS) (14). In these patients, oxygenation index improved following hemoadsorption therapy, as follows: Patient three: from 11 to 9; Patient four: from 18 to 17, Patient five: from 31 to 23, Patient six: from 23 to 6, Patient seven: from 16 to 20, Patient nine: from 25 to 22 (Table 2).

### Hematological and liver profile

No statistically significant changes were observed in leukocyte or platelet counts, nor for total bilirubin levels. Bilirubin remained stable, with a median pre- and post-intervention level of 1.02 and 1.05 mg/dL, respectively (*p* = 1.00). One neonatal patient with cholestasis and a direct bilirubin of 76 mg/dL showed a decrease to 8.2 mg/dL with hemoadsorption therapy. However, in patients with mild hyperbilirubinemia, we did not observe any clinically or statistically significant changes.

### Mortality and clinical outcomes

In-hospital mortality was 54.5% (*n* = 6). Deceased patients showed less reduction in VIS and lactate compared to survivors, although statistical significance was not reached. The median PICU stay was 14 days (IQR: 11–23), and the median total hospital stay was 23 days (IQR: 11–35).

## Discussion

In this retrospective study of 11 critically ill neonatal and pediatric patients, we evaluated the clinical and laboratory impact of hemoadsorption therapy using CytoSorb^®^ or Oxiris^®^ in combination with extracorporeal life support modalities (CRRT and/or ECMO). Our findings suggest that hemoadsorption may contribute to hemodynamic stabilization, improved tissue perfusion, attenuation of the inflammatory response, and enhanced oxygenation—particularly in the context of refractory septic shock and multiple organ dysfunction syndrome (MODS). These results provide valuable insights into the potential utility, safety and limitations of hemoadsorption in a resource-limited, real-world pediatric ICU setting.

While our data support the potential utility of hemoadsorption in this clinical context, it is critical to interpret these findings within the framework of a resource-limited Latin American setting. Rather than confirming previously reported effects, our cohort provides preliminary, context-specific insight into feasibility, safety, and response patterns, paving the way for protocol development, cost-effectiveness evaluations, and multicenter collaboration in similar healthcare settings. The implementation of advanced extracorporeal therapies, such as CRRT, ECMO, and hemoadsorption, is associated with logistical and infrastructural challenges, including high costs, limited availability of devices and consumables, and a scarcity of specialized personnel. In this context, the use of hemoadsorption must be judicious, driven by strong physiological rationale and real-time clinical response rather than only protocolized algorithms derived from high-resource settings. As such, our study, although small, demonstrates that even in constrained environments, it is possible to deliver technically complex interventions safely, provided that clear physiological targets are used to guide therapy. The adjunctive use of hemoadsorption may offer a pragmatic option in the armamentarium of tertiary care centers in low- and middle-income countries, particularly for patients with otherwise refractory shock or hyperinflammatory states. However, the therapeutic window for benefit is likely narrow, and delays in access or lack of precision in patient selection may limit efficacy or even pose harm.

Hemoadsorption is increasingly used as an adjunctive therapy in critical care, with expanding evidence supporting its technical feasibility and immunomodulatory potential. Recent comprehensive reviews have outlined the current state of the art in hemoadsorption, highlighting its ability to remove a wide range of inflammatory mediators via sorbent-based cartridges integrated into extracorporeal circuits (15, 16). As such, cytokine removal is considered a key mechanistic rationale for hemoadsorption therapy in patients with sepsis and multiple organ dysfunction, where excessive or dysregulated immune responses play a central role. Non-selective hemoadsorption devices, including CytoSorb^®^ and Oxiris^®^, are designed to target circulating inflammatory mediators within the intermediate molecular weight range (up to approx. 60 kDa), including key cytokines such as interleukin (IL)-6, IL-10, tumor necrosis factor-alpha (TNF-*α*), interferon-gamma (IFN-*γ*), and monocyte chemoattractant protein-1 (MCP-1). Proof-of-concept for this approach was established in a foundational study by Jansen et al., demonstrating that CytoSorb hemoperfusion markedly attenuates circulating cytokine concentrations during systemic inflammation in humans *in vivo* (17).

Recent pediatric data corroborate these mechanistic expectations. Bottari and colleagues provided pediatric-specific evidence from the PedCyto study, showing potential correlations between immune modulation and improved hemodynamic stability in children undergoing hemoadsorption with CytoSorb^®^ and CRRT (18). In a prospective study by Hui et al. involving 11 pediatric cases of hemoadsorption, a ≥ 50% reduction was achieved in 60.5% of cytokines assessed, with a more pronounced and sustained decline observed in survivors (19). Similarly, Rihar et al. reported clinical improvement and reductions in IL-6 in children >10 kg treated with CytoSorb^®^ in the setting of a cytokine storm due to sepsis, showing an association between biochemical response and positive clinical outcomes (20). While not directly comparable to our cohort, a case series by Sánchez-Morán et al. described the use of hemoadsorption combined with CRRT in adult patients with abdominal sepsis, using a different device, and reported positive effects on inflammatory biomarkers, hemodynamics and clinical parameters, further supporting the rationale for extracorporeal cytokine modulation in septic hyperinflammatory states (21).

The ADQI 30 consensus acknowledges the ability of these devices to reduce cytokine burden, particularly when applied during the early hyperinflammatory phase of critical illness. However, it also emphasizes the urgent need for biomarker-driven approaches to individualize therapy initiation and monitor response (8).

Although our study did not directly measure cytokine concentrations, we observed indirect evidence of inflammatory modulation. A substantial reduction in C-reactive protein (CRP) levels (34 → 11 mg/L; −67.6%) was recorded, though this did not reach statistical significance (*p* = 0.083). A significant reduction in serum lactate (4.7 → 2.5 mmol/L; *p* < 0.001), accompanied by an increase in pH (7.28 → 7.43; *p* = 0.004), was observed following treatment. These improvements are likely attributable to the effects of CRRT, which contributes to acid–base correction and lactate clearance. However, it is also plausible that hemoadsorption played a contributory role by supporting hemodynamic stabilization and modulating the inflammatory response. Additionally, the relatively short duration of hemoadsorption in our cohort (less than 48 h in most cases) may have limited the extent of measurable metabolic effects, potentially contributing to the observed patterns. Given the combined use of extracorporeal therapies in most patients, the individual contribution of each modality cannot be fully delineated.

In states of systemic inflammation, particularly in septic shock, vasoactive-inotropic support is often required to maintain adequate perfusion, yet prolonged exposure to high doses carries substantial risk, including ischemia, and impaired end-organ perfusion. Thus, interventions that can modulate the inflammatory cascade and reduce vasopressor burden are of considerable therapeutic interest. In our cohort, we observed a significant reduction in both the PELOD-2 score (from median 11 to 7; *p* = 0.036) and the Vasoactive-Inotropic Score (VIS) (from median 75 to 6; *p* = 0.014), reflecting improvements in both global organ dysfunction and cardiovascular stability. This finding is in line with Rihar et al., who also observed hemodynamic stabilization in their study population (20). The magnitude of VIS reduction (>90%) suggests a robust modulation of the inflammation–vasopressor axis. This aligns with findings by Bottari et al., who, in a secondary analysis of the NCT05658588 study, reported similar reductions in PELOD-2 and VIS scores within 72–96 h of initiating hemoadsorption in combination with CRRT (22). Importantly, these improvements were accompanied by recovery of sublingual microcirculation, as measured by mean flow index (MFI) and proportion of perfused vessels (PPV) using videomicroscopy techniques (both >90%), potentially reflecting not only improved perfusion but also enhanced global metabolic status and highlighting the potential systemic benefit of cytokine removal in restoring vascular homeostasis.

In addition to its immunomodulatory and hemodynamic effects, hemoadsorption may offer benefits in respiratory function, particularly in the setting of sepsis- or ECMO-associated acute respiratory distress syndrome (ARDS). In our cohort, we observed a statistically significant improvement in oxygenation, reflected by an increase in the PaO₂/FiO₂ ratio from 69 to 104 mmHg (*p* = 0.042), and a concurrent 50% reduction in the oxygenation index, a parameter inversely associated with pulmonary compliance and gas exchange efficiency. This finding is also supported by studies in pediatric patients with ARDS (19), as well as systematic reviews in adults with various etiologies, in which hemoadsorption has shown improvement in pulmonary mechanics and oxygenation parameters, albeit with heterogeneous results (23).

Hematological parameters, including leukocyte and platelet counts, remained stable, indicating the biocompatibility of the sorbent devices used. This aligns with the ADQI 30 consensus, which highlights theoretical risks of cellular adsorption, particularly in smaller children or with less biocompatible cartridges (8). While bilirubin levels remained largely unchanged across the cohort, one neonate with marked cholestasis demonstrated a substantial decline in direct bilirubin levels following hemoadsorption, consistent with observations by Hui et al. in cases of severe hyperbilirubinemia (19). This response reflects the concentration-dependent adsorption kinetics of the CytoSorb cartridge, where higher substrate concentrations promote more efficient removal most probably due to steeper concentration gradients between blood and the sorbent surface. As levels decrease, the adsorption rate diminishes accordingly—not necessarily as a result of cartridge saturation, but due to reduced driving force for molecular transfer. Overall these findings suggest that hemoadsorption may also offer benefit in selected patients with hepatic dysfunction.

While the clinical application of hemoadsorption has shown promise in modulating inflammation and supporting organ function, its integration into pediatric critical care requires careful attention to pharmacokinetic implications—particularly in relation to antimicrobial therapy. Bottari et al. recently reported significant reductions in serum levels of antimicrobials, including levofloxacin and ceftazidime, during hemoadsorption therapy, while showing a low clearance for amikacine and higher mass removal and clearance with CRRT compared to CytoSorb. Still, this concern is especially pertinent in pediatric patients, who typically exhibit smaller volumes of distribution, higher metabolic rates, and more pronounced hemodynamic instability than adults. These factors increase their susceptibility to rapid pharmacokinetic fluctuations during extracorporeal therapies. Consequently, individualized antimicrobial dosing and close therapeutic drug monitoring (TDM) should be considered standard practice when hemoadsorption is used in this population—particularly for time- or concentration-dependent antibiotics with narrow therapeutic windows.

In parallel, both the ADQI 30 consensus (8) and the Indian expert consensus (24) emphasize the critical importance of early initiation of hemoadsorption to maximize its therapeutic benefit. Specifically, initiation beyond 24–48 h after the onset of shock or clinical deterioration is associated with diminished efficacy, possibly due to progression to an immunosuppressive phase or irreversible organ injury. This recommendation, coupled with prior consensus statements and pharmacokinetic concerns, supports a more proactive, protocolized approach to hemoadsorption—ideally integrated into early goal-directed therapy algorithms for sepsis and MODS. Notably, all patients weighing less than 10 kg in the study by Rihar et al., as well as in our cohort, did not survive (20), which points toward the fact that body weight could be an indirect predictor of tolerance of the extracorporeal volumes associated with extracorporeal therapies. However, this observation also highlights the urgent need to adapt hemoadsorption technology and protocols for safe and effective use in neonates and infants.

In our cohort, in-hospital mortality was 54.5%, aligning with previously reported outcomes in similarly critically ill pediatric populations undergoing hemoadsorption such as Hui et al., reporting a 45% mortality rate in their pediatric cohort. Interestingly, within our study, non-survivors exhibited a markedly lower reduction in vasoactive-inotropic score (VIS) and serum lactate, compared to survivors. While these observations did not reach statistical significance, they suggest a potential role for VIS and lactate clearance as early surrogate markers of clinical response and prognosis. Given that both reflect circulatory function and tissue perfusion, their trajectory may provide real-time feedback on the hemodynamic and metabolic impact of hemoadsorption.

### Limitations

This study has several important methodological limitations that warrant consideration. Most notably, the small sample size substantially limits statistical power and constrains the generalizability of findings beyond our single-center context. The retrospective and uncontrolled design precludes adjustment for confounding variables and does not allow for multivariate analysis or survival modeling, thereby limiting our ability to draw causal inferences between hemoadsorption and observed clinical improvements. Furthermore, all patients were already receiving advanced extracorporeal support (ECMO and/or CRRT) prior to the initiation of hemoadsorption, introducing a potential selection bias toward more severely ill individuals. The heterogeneity of underlying pathologies—ranging from septic shock to toxicological emergencies—further complicates interpretation, as it becomes challenging to disentangle the independent effect of hemoadsorption from that of the underlying disease process and concurrent therapies. Despite these limitations, this study provides valuable exploratory data and reinforces the feasibility of hemoadsorption as an adjunctive intervention in pediatric critical care, particularly in resource-limited settings. The preliminary signals of benefit observed here underscore the urgent need for well-powered, prospective, and multicenter trials, ideally incorporating biomarker-guided inclusion criteria and standardized outcome measures.

## Conclusion

This study, conducted in a Latin American setting with limited healthcare resources, provides important preliminary evidence supporting the feasibility and potential clinical utility of hemoadsorption therapy in critically ill pediatric and neonatal patients, particularly when used in conjunction with CRRT and/or ECMO. Hemoadsorption was associated with improvements in key clinical parameters, including hemodynamic stability, inflammatory burden, and organ dysfunction. However, its effect on mortality remains uncertain and likely depends on multiple interrelated factors. Notably, outcomes in neonates and low-weight infants were poor, underscoring the urgent need for pediatric-specific device adaptations and protocol development to optimize safety and efficacy in this vulnerable subgroup. Future research should focus on defining optimal timing, patient selection criteria, and biomarker-guided approaches, as well as evaluating pharmacokinetic considerations unique to the pediatric population.

## Data Availability

The original contributions presented in the study are included in the article/supplementary material, further inquiries can be directed to the corresponding author.

## References

[1] Levin DL Downes JJ Todres ID. History of pediatric critical care medicine. J Pediatr Intensive Care. (2013) 2:147–67. doi: 10.3233/PIC-13068 31214438 PMC6530732

[2] Dendir G Awoke N Alemu A Sintayhu A Eanga S Teshome M. Factors associated with the outcome of a Pediatric patients admitted to intensive care unit in resource-limited setup: cross-sectional study. Pediatric Health Med Ther. (2023) 14:71–9. doi: 10.2147/PHMT.S389404, PMID: 36890923 PMC9987449

[3] Slusher TM Kiragu AW Day LT Bjorklund AR Shirk A Johannsen C. Pediatric critical care in resource-limited settings-overview and lessons learned. Front Pediatr. (2018) 6:49. doi: 10.3389/fped.2018.00049, PMID: 29616202 PMC5864848

[4] Miranda M Nadel S. Pediatric Sepsis: a summary of current definitions and management recommendations. Curr Pediatr Rep. (2023) 11:29–39. doi: 10.1007/s40124-023-00286-3, PMID: 37252329 PMC10169116

[5] Totapally A Bridges BC Selewski DT Zivick EE. Managing the kidney - the role of continuous renal replacement therapy in neonatal and pediatric ECMO. Semin Pediatr Surg. (2023) 32:151332. doi: 10.1016/j.sempedsurg.2023.151332, PMID: 37871460

[6] Erdil T Lemme F Konetzka A Cavigelli-Brunner A Niesse O Dave H. Extracorporeal membrane oxygenation support in pediatrics. Ann Cardiothorac Surg. (2019) 8:109–15. doi: 10.21037/acs.2018.09.08, PMID: 30854319 PMC6379197

[7] Canter MO Daniels J Bridges BC. Adjunctive therapies during extracorporeal membrane oxygenation to enhance multiple organ support in critically ill children. Front Pediatr. (2018) 6:78. doi: 10.3389/fped.2018.00078, PMID: 29670870 PMC5893897

[8] Bellomo R Ankawi G Bagshaw SM Baldwin I Basu R Bottari G. Hemoadsorption: consensus report of the 30th acute disease quality initiative workgroup. Nephrol Dial Transplant. (2024) 39:1945–64. doi: 10.1093/ndt/gfae089, PMID: 38621759

[9] Waalders NJB Kox M Pickkers P. Haemoadsorption to remove inflammatory mediators in sepsis: past, present, and future. Intensive Care Med Exp. (2025) 13:38. doi: 10.1186/s40635-025-00740-0, PMID: 40117010 PMC11928715

[10] Mitzner S Kogelmann K Ince C Molnár Z Ferrer R Nierhaus A. Adjunctive hemoadsorption therapy with CytoSorb in patients with septic/Vasoplegic shock: a best practice consensus statement. J Clin Med. (2023) 12:7199. doi: 10.3390/jcm12237199, PMID: 38068250 PMC10707447

[11] Broman ME Hansson F Vincent J-L Bodelsson M. Endotoxin and cytokine reducing properties of the oXiris membrane in patients with septic shock: a randomized crossover double-blind study. PLoS One. (2019) 14:e0220444. doi: 10.1371/journal.pone.0220444, PMID: 31369593 PMC6675097

[12] Bottari G Guzzo I Cappoli A Labbadia R Perdichizzi S Serpe C. Impact of CytoSorb and CKRT on hemodynamics in pediatric patients with septic shock: the PedCyto study. Front Pediatr. (2023) 11:1259384. doi: 10.3389/fped.2023.1259384, PMID: 37780052 PMC10540853

[13] Morin L Ray S Wilson C Remy S Benissa MR Jansen NJG. Refractory septic shock in children: a European Society of Paediatric and Neonatal Intensive Care definition. Intensive Care Med. (2016) 42:1948–57. doi: 10.1007/s00134-016-4574-2, PMID: 27709263 PMC5106490

[14] Emeriaud G López-Fernández YM Iyer NP Bembea MM Agulnik A Barbaro RP. Executive summary of the second international guidelines for the diagnosis and management of pediatric acute respiratory distress syndrome (PALICC-2). Pediatr Crit Care Med. (2023) 24:143–68. doi: 10.1097/PCC.0000000000003147, PMID: 36661420 PMC9848214

[15] Chang K Li Y Qin Z Zhang Z Wang L Yang Q. Effect of extracorporeal hemoadsorption in critically ill patients with COVID-19: a narrative review. Front Immunol. (2023) 14:1074465. doi: 10.3389/fimmu.2023.1074465, PMID: 36817416 PMC9936071

[16] Ronco C Bellomo R. Hemoperfusion: technical aspects and state of the art. Crit Care. (2022) 26:135. doi: 10.1186/s13054-022-04009-w, PMID: 35549999 PMC9097563

[17] Jansen A Waalders NJB van Lier DPT Kox M Pickkers P. CytoSorb hemoperfusion markedly attenuates circulating cytokine concentrations during systemic inflammation in humans *in vivo*. Crit Care. (2023) 27:117. doi: 10.1186/s13054-023-04391-z, PMID: 36945034 PMC10029173

[18] Bottari G Cecchetti C Serpe C Grimaldi D Taccone FS. Potential correlation between hemodynamic improvement and an immune-modulation effect in pediatric patients with septic shock treated with renal replacement therapy and CytoSorb®: an insight from the PedCyto study. Crit Care. (2024) 28:25. doi: 10.1186/s13054-024-04802-9, PMID: 38233883 PMC10792820

[19] Hui WF Chan RWY Wong CK Cheung WL Ku SW Hon KL. The pattern of cytokine profile in children received extracorporeal blood purification. Int J Artif Organs. (2025) 48:123–9. doi: 10.1177/03913988251313885, PMID: 39851190

[20] Rihar E Peršič V Jerman A Plankar Srovin T Mlakar G Bezeljak N. Hemoperfusion with CytoSorb® in Pediatric patients: a monocentric case series. J Clin Med. (2024) 13:6587. doi: 10.3390/jcm13216587, PMID: 39518726 PMC11547108

[21] Sánchez-Morán F Mateu-Campos ML Bernal-Julián F Gil-Santana A Sánchez-Herrero Á Martínez-Gaspar T. Haemoadsorption combined with continuous renal replacement therapy in abdominal sepsis: case report series. J Pers Med. (2023) 13:1113. doi: 10.3390/jpm13071113, PMID: 37511726 PMC10381379

[22] Bottari G Confalone V Creteur J Cecchetti C Taccone FS. The sublingual microcirculation in critically ill children with septic shock undergoing Hemoadsorption: a pilot study. Biomedicine. (2024) 12:1435. doi: 10.3390/biomedicines12071435, PMID: 39062009 PMC11275152

[23] Szigetváry CE Turan C Kovács EH Kói T Engh MA Hegyi P. Hemoadsorption as adjuvant therapy in acute respiratory distress syndrome (ARDS): a systematic review and meta-analysis. Biomedicine. (2023) 11:3068. doi: 10.3390/biomedicines11113068, PMID: 38002070 PMC10669540

[24] Mehta Y Ansari AS Mandal AK Chatterjee D Sharma GS Sathe P. Systematic review with expert consensus on use of extracorporeal hemoadsorption in septic shock: an Indian perspective. World J Crit Care Med. (2024) 13:89026. doi: 10.5492/wjccm.v13.i1.89026, PMID: 38633478 PMC11019629

